# The Heart in Acute Lobar Pneumonia

**Published:** 1904-07-02

**Authors:** 


### THE HEART IN ACUTE LOBAR PNEUMONIA.

In an analysis of 200 cases of acute lobar pneumonia, Dr. Jno. Hay[1] makes some interesting remarks regarding the significance of the circulatory symptoms as bearing upon prognosis and treatment. Cardiac failure is signalled by pallor of the face, poorly filled capillaries, and low delirium. Increasing rate of the respirations, and especially the appearance of moist rales in the sound lung, tell a similar tale, and must be regarded as carrying serious significance. Extension of the area of cardiac dulness, especially towards the right, weakening of the cardiac sounds, the first at the mitral and tricuspid areas and the second at the pulmonic cartilage, are amongst the earliest signs of danger from cardiac failure. In regard to the pulse—a very soft pulse with a poor percussion wave and the sphygmograph showing only a slight dicrotic wave, form in the early stage of the disease a combination of events suggesting a feeble state of the myocardium and a correspondingly anxious prognosis. The rate of the pulse may vary within wide limits, but 130 or more per minute in an adult, and sustained for 48 hours or upwards, is generally associated with a fatal issue. A falling pulse usually precedes by some hours the typical sudden fall of temperature which marks the "crisis" and is therefore of good omen. On the other hand, increased pulse and respiration rates, even with a falling thermometer, is a bad sign. Irregularity of the pulse is not common in pneumonia, not even in fatal cases, except in the immediately ante-mortem period. It may, however, occur at any period of the illness, and is of least moment in the old, or at the crisis. Dr. Hay is able to quote six cases of cardiac irregularity before the crisis in which the patients recovered, though he agrees that this condition is usually followed by a fatal issue.

To prevent cardiac failure, in addition to the necessity for quiet and peaceful surroundings, it must be remembered that influences tending to determine such failure, are sleeplessness, struggling associated with delirium, and flatulent distension of the abdomen. Insomnia, in the early stages, may be met by such simple measures as cold and tepid sponging, and by relief of pain by poultices or ice-bags; paraldehyde in doses of 2 to 3 drachms is sometimes useful. In reference to the use of opium in pneumonia Dr. Hay takes a very decided position. As will be remembered, strong views, both for and against, have been expressed by such authorities as Sir Samuel Wilks on the one side, and Sir William Gairdner on the other. Our present author claims that when the above measures fail the best drug is opium, and he prefers to other methods of administration the hypodermic use of morphine—that is the method which the opponents of opium in pneumonia regard as particularly dangerous. For himself, he considers that before the fifth day there is rarely need for hesitation. His argument, apart from the fact of successful practice, is that the risk of morphine is less than the danger of active and struggling delirium, and that in this condition morphine is the only drug on which reliance can be placed. Evidences of cardiac failure, of diffused bronchitis, of œdema of the lungs, or of kidney disease are to be recognised as forbidding the use of opium.

Abdominal distension prejudices the action of the heart, both by direct pressure and by preventing free movement of the diaphragm, thus hindering respiratory movement in carrying on the pulmonary circulation and increasing the burden of the right ventricle. When due to flatulence it generally means over-ingestion of milk, and needs cutting down the food supply, a dose of calomel, and such an antiseptic as glycerine of carbolic acid. Distension due to toxæmia may be partly met by calomel and enemata, but calls also for general measures. When evidences of cardiac failure appear the claim is for such remedies as strychnine, digitalis, caffeine and ammonium carbonate, that is for cardiac stimulants and tonics, not for alcohol, which is a sedative and tends to produce vaso-motor paralysis and vascular engorgement. Both strychnine and digitalis are best administered hypodermically. Ammonium carbonate is particularly serviceable in cases where there is much bronchitis; it should be given in 10-grain doses every four hours, with occasional doses of 20 to 30 grains in addition. It rarely causes emesis and is usually followed by very free expectoration. Lastly, Dr. Hay favours the application of ice-bags to the abdomen as recommended by Dr. Barr, of Liverpool. Care should be taken to prevent the ice-bag slipping down so as to come into contact with the groins, as this causes the patient much discomfort.

[1] Lancet, June 11, 1904.

